# Binary Morphological Filtering of Dominant Scattering Area Residues for SAR Target Recognition

**DOI:** 10.1155/2018/9680465

**Published:** 2018-12-03

**Authors:** Chao Shan, Bin Huang, Minggao Li

**Affiliations:** ^1^Centre of Nautical and Aviation Medicine of the PLA, Navy General Hospital, Beijing 100048, China; ^2^Department of Electronic Engineering, Tsinghua University, Beijing 100084, China

## Abstract

A synthetic aperture radar (SAR) target recognition method is proposed in this study based on the dominant scattering area (DSA). DSA is a binary image recording the positions of the dominant scattering centers in the original SAR image. It can reflect the distribution of the scattering centers as well as the preliminary shape of the target, thus providing discriminative information for SAR target recognition. By subtracting the DSA of the test image with those of its corresponding templates from different classes, the DSA residues represent the differences between the test image and various classes. To further enhance the differences, the DSA residues are subject to the binary morphological filtering, i.e., the opening operation. Afterwards, a similarity measure is defined based on the filtered DSA residues after the binary opening operation. Considering the possible variations of the constructed DSA, several different structuring elements are used during the binary morphological filtering. And a score-level fusion is performed afterwards to obtain a robust similarity. By comparing the similarities between the test image and various template classes, the target label is determined to be the one with the maximum similarity. To validate the effectiveness and robustness of the proposed method, experiments are conducted on the moving and stationary target acquisition and recognition (MSTAR) dataset and compared with several state-of-the-art SAR target recognition methods.

## 1. Introduction

As a microwave sensor, the synthetic aperture radar (SAR) could operate under all-day and all-weather conditions with the capability to penetrate the shelters such as clouds and trees. Therefore, it is widely used in both military and civilian fields. With the fast development of SAR sensors, the interpretation of high-resolution SAR images has been an urgent task. As one branch of SAR image interpretation, automatic target recognition (ATR) aims to decide the target class of an unknown region of interest (ROI) obtained by target detection [1]. Since the 1990s, SAR ATR has been studied intensively with a rich set of effective methods. Generally, the SAR ATR methods can be categorized into two types: template-based ones [2, 3] and model-based ones [4, 5]. The two categories differ in the ways of describing the characteristics of the targets. The template-based systems store the SAR images from different conditions such as different view angles and backgrounds to describe the characteristics of the targets. In contrast, the model-based methods use the physical or conceptual models to describe the targets, such as CAD models [4, 5] or 3D scattering center models [6, 7]. For a concrete SAR ATR algorithm, both the template-based and model-based methods involve two steps: feature extraction and classification. The former step is performed to obtain discriminative representations for the original SAR images. In the field of SAR ATR, different kinds of features are used including the geometrical features [8–13], transformation features [14–18], and scattering center features [19–25]. The geometrical features are extracted to describe the geometrical properties of the target. In [9, 10], the Zernike and Krawtchouk moments are adopted to describe the binary target regions, respectively. Afterwards, the resulted feature vectors are classified by support vector machines (SVMs). The outline points of the target are extracted and approached by the elliptical Fourier series (EFS) in [12]. Similarly, the extracted descriptors are classified by SVM to determine the target label. As the product of the target and background, the shadow reflects the target's profile indirectly. Hence, Papson and Narayanan model the shadow outline using the hidden Markov model (HMM) with application to target recognition [13]. The transformation features are also frequently used in SAR ATR. These features can be extracted with notably high efficiency by using methods such as principal component analysis (PCA) [14], linear discriminant analysis (LDA) [14], and nonnegative matrix factorization (NMF) [15]. Afterwards, the feature vectors with unified forms can be efficiently classified by the advanced classifiers like SVM and sparse representation-based classification (SRC). In [14], PCA and LDA are used for SAR feature extraction, which are classified by K-nearest neighbor (KNN). In [15], NMF is introduced into SAR ATR, which achieves superior performance than LDA and PCA according to the experimental results. Inspired by the manifold learning, some other transformation features are designed to improve the ATR performance [16–18]. The scattering center features mainly describe the electromagnetic scattering phenomenon of the target. As a typical representative, the attributed scattering center has been applied to SAR ATR with good performance. In [21–24], several matching schemes are proposed to build one-to-one correspondences between two attributed scattering center sets. Afterwards, similarity measures are defined for target recognition. In [6], the scattering centers are used in a model-based way, where the predicted scattering centers by 3D scattering centers are matched with the extracted target region or scattering centers. In the classification stage, the classifier is designed according to the properties of the features. Owing to the fast development in the field of computer vision, many advanced classifiers have been successfully applied to SAR target recognition with good performance including SVM [9, 12, 26, 27], adaptive boosting (AdaBoost) [28], SRC [27, 29, 30], and discriminative graphical models [31]. Recently, the deep learning method, i.e., convolutional neural network (CNN), is demonstrated to be effective for SAR ATR [32–36]. CNN learns hierarchical features by the convolution layers and performs target classification via a softmax layer (i.e., a multiclass regression analysis) [37, 38]. However, for features with no unified forms, e.g., unordered scattering centers, the traditional nearest neighbor is often adopted. In detail, a similarity (distance) measure is first designed for the features, and the target label is decided to be the template class (model) with the maximum similarity (minimum distance) [21–24].

In this paper, an SAR ATR method is proposed based on the dominant scattering area (DSA) [39]. DSA is generated by selecting several dominant scattering points with the highest intensities in the original SAR images. In this way, DSA reflects the spatial distribution of the dominant scattering centers. Meanwhile, the preliminarily geometrical shape of the target can also be described. Therefore, DSA could provide rich discriminability for SAR ATR. In the classification stage, the DSA of the test sample is matched with the DSAs of its corresponding templates from different classes. The DSA residues between the test sample and its correct template are small patches. In contrast, when the template is from the incorrect classes, the residues are usually bulkily shaped with large areas. In this case, the target label can be correctly determined based on the areas of the DSA residues with high probabilities. However, the test image may be corrupted severely by the extended operating conditions (EOCs) like noise corruption and partial occlusion. As a result, its DSA may be partially deformed. To handle these situations, the binary morphological filtering, i.e., the opening operation [40], is performed on the DSA residues to further enhance their discriminability in this study. After the opening operation, the filtered DSA residues between the intraclass samples are largely narrowed, whereas the majority of the between-class residues are retained. So, it is easier to distinguish different targets based on the filtered DSA residues. A similarity measure is defined based on the filtered DSA residues after the opening operation, which comprehensively considers the distribution properties of the DSA residues and possible deformations of DSA. Moreover, to handle unpredictable deformations of the test sample, several structuring elements are jointly used during the morphological filtering. Afterwards, a score-level fusion is performed to combine the results at different structuring elements to improve the robustness of the similarity measure. Finally, the target class of the test sample is decided according to the fused similarities.

The remainder of this paper is organized as follows: Section 2 introduces the fundamentals of the binary morphological filtering. In Section 3, the target recognition method based on the DSA is presented. Experiments are conducted on the moving and stationary target acquisition and recognition (MSTAR) dataset in Section 4. Conclusions are drawn in Section 5 with some discussions.

## 2. Binary Morphological Filtering

The binary morphological filtering [40] processes the binary images with the structuring elements, which can modify the distributions and shapes of the binary regions. As key to binary morphological filtering, the structuring element greatly influences the filtered results. During the filtering, the structuring element slides on the binary image and returns a binary result based on set theory.

Erosion and dilation are two basic operations in binary morphological filtering. For a binary image *I*, its corresponding results after the erosion and dilation operations are obtained as follows:

(1) $$\begin{array}{c} E=I\;⊖\;S=\left\{\left.x,y\;\right|{\;S}_{xy}\;\subseteq \;B\right\},\\ D=I\;⊕\;S=\left\{\left.x,y\;\right|{\;S}_{xy}\;\cap \;B=\emptyset \right\}, \end{array}$$

where the symbols ⊖ and ⊕ represent the erosion and dilation operations, respectively; *S* denotes the structuring element; and *E* and *D* are the results after the erosion and dilation, respectively. The erosion operation can effectively remove the regions with smaller sizes than the structuring element. In contrast, the dilation operation can enlarge the binary regions according to the structuring element.

According to the properties, the other two binary morphological filtering methods, i.e., opening and closing operations, are designed as follows:

(2) $$\begin{array}{c} I\circ S=\left(I⊖S\right)⊕S,\\ I•S=\left(I⊕S\right)⊖S, \end{array}$$

where the symbols ∘ and • represent the opening and closing operations, respectively. In the opening operation, the binary region is first filtered using the erosion operation and the dilation operation afterwards. The closing operation works in the opposite order. According to the properties of the erosion and dilation operations, the opening operation is capable of breaking the narrow connections and smoothing the region outlines. Differently, the closing operation can eliminate the holes to connect the whole binary region.

## 3. DSA Matching Using Morphological Operations

### 3.1. Generation of Dominant Scattering Area (DSA)

SAR images reflect the backscattering of the target. The pixels with higher intensities in SAR images indicate they have stronger scatterings. By selecting a certain number of the strongest scattering points, a binary DSA image can be generated. The procedure of generating DSA is as follows:

***Step 1*.**

Sort the intensities of the original image in the descending manner

***Step 2*.**

Decide the threshold for segmentation based on the number of desired dominant scattering points

***Step 3*.**

Convert the original SAR image to a binary image according to the threshold in Step 2.

**Figure 1:**
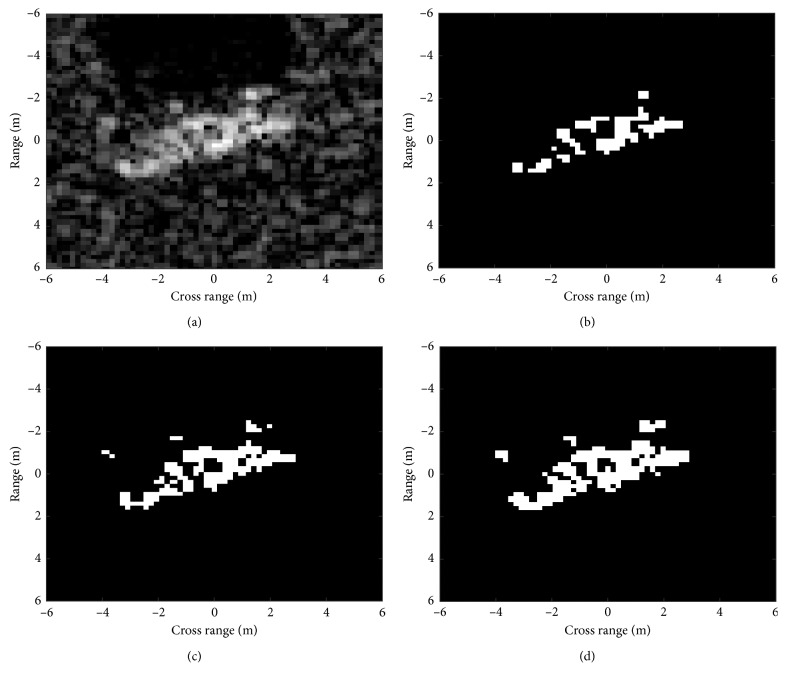
The DSAs generated at different numbers of dominant scattering points: (a) original image; (b) DSA at 100 dominant scattering points; (c) DSA at 150 dominant scattering points; (d) DSA at 200 dominant scattering points.

The generation of DSA is notably simple and convenient compared with the traditional target segmentation algorithms. Figures 1(b)–1(d) show the DSAs of the original image in Figure 1(a) by selecting different numbers of scattering points at 100, 150, and 200, respectively. It is visible that the DSAs can reflect the distribution of the scattering centers as well as the preliminary target shape. The number of selected scattering points is also crucial. Too few selections will deform the target shape, while a large number of scattering points will bring too many false alarms from the background clutters. According to the observations of many MSTAR images, the choice of selecting 150 dominant scattering points is relatively robust for this special dataset. In comparison with the binary target region, the generated DSAs have the following advantages: first, it is much easier to generate the DSA than the binary target region. As illustrated above, the steps for generating the DSA are very simple and efficient. The main reason is that the dominant scattering centers have much higher intensities than the background pixels. However, for the binary target region, some weak scattering centers on the target may have approaching intensities with the background pixels, which makes it difficult for the precise target segmentation. Second, as shown in Figure 1, the DSA can not only describe the distribution of the scattering centers but also reflect the geometrical shape of the target. Therefore, the DSA can provide more discriminative information than the binary target region, which mainly depicts the geometrical shape of the target. Third, in this study, the multilevel DSAs are jointly used. As shown in Figure 1, they provide coarse-to-fine descriptions of the target characteristics. Therefore, it is promising that their joint classification can promote the recognition performance.

### 3.2. DSA Matching Using Binary Morphological Filtering

#### 3.2.1. Similarity Measure Based on DSA Residue

DSA reflects the distribution of the scattering centers as well as the preliminary target shape. Therefore, the DSA residues actually reflect the differences in the two images. From this aspect, the DSA residues can be effectively used to distinguish different classes of targets. Denote the DSAs generated from the test image and its corresponding template image as *F* and *G*, respectively; the DSA residues *R* are obtained by a simple subtraction as follows:

(3) $$\begin{array}{c} R=\left|F-G\right|. \end{array}$$

**Figure 2:**
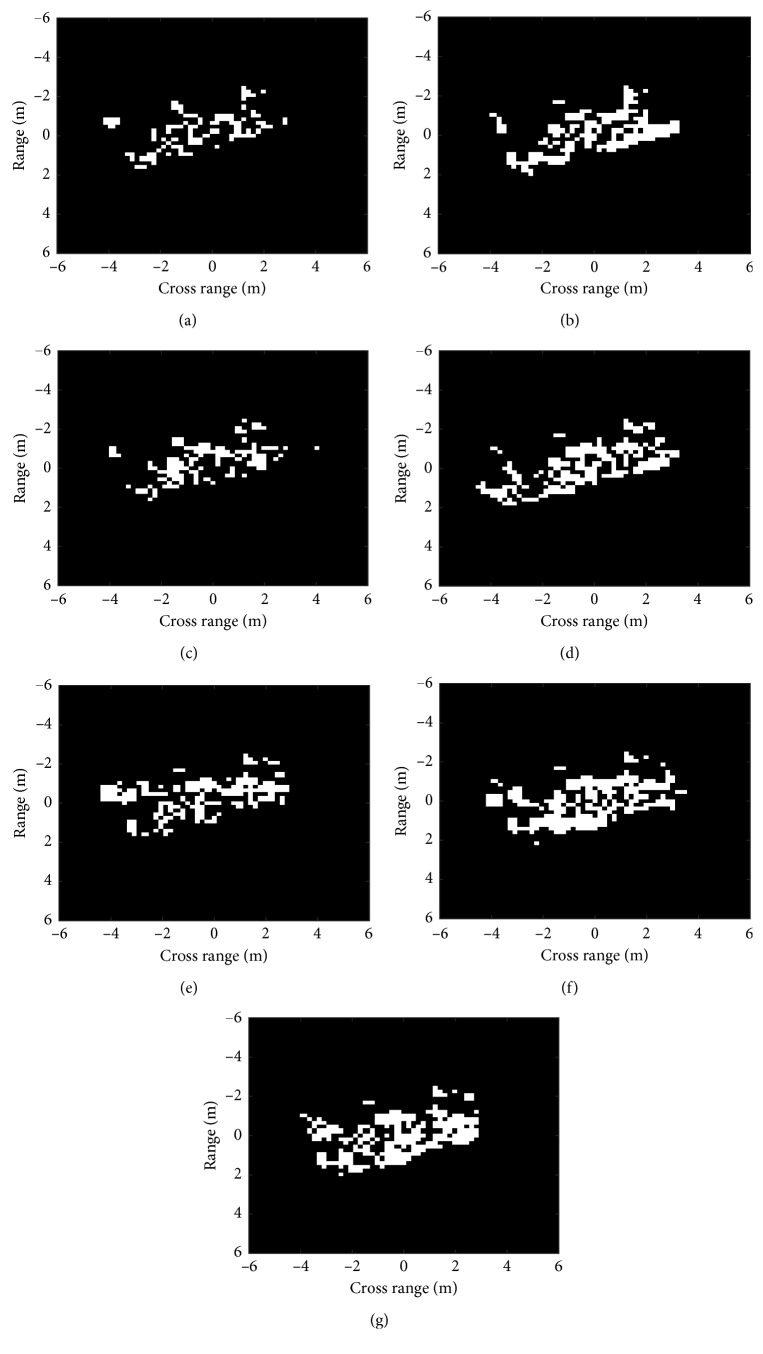
DSA residues between a BMP2 image and its corresponding template images from different classes: (a) BMP2 (Sn_9563); (b) BMP2 (Sn_9566); (c) BMP2 (Sn_c21); (d) BTR70; (e) T72 (Sn_132); (f) T72 (Sn_812); (g) T72 (Sn_s7).

Figure 2 shows the DSA residues between a BMP2 image and its corresponding images at the same azimuth in the template database comprised by BMP2, BTR70, and T72 (the detailed descriptions of these targets are presented in Section 4). Clearly, the DSA residues with the correct class have much less nonzero elements than those with the incorrect classes. Denote the areas of the nonzero regions (i.e., the numbers of nonzero pixels) in *F*, *G*, and *R* as *N*_*F*_, *N*_*G*_, and *N*_*R*_, respectively; the similarity between *F* and *G* is defined as follows:

(4) $$\begin{array}{c} C_{F,G}=1-\frac{N_{R}}{N_{F}+N_{G}}. \end{array}$$

Equation (4) gives a normalized similarity. When the DSAs of two images totally overlap, the similarity is 1, and the similarity equals 0 when the two DSAs have no overlaps. Ideally, for a test image, the largest similarity is obtained with its corresponding template from the same class. And the similarities with the incorrect classes are relatively lower. Then, the test image can be recognized correctly. However, due to the possible corruptions of the DSAs caused by EOCs like noise contamination and occlusion, it is inadequate to make reliable judgments just using the original DSA residues. Therefore, the discriminability in the DSA residues should be further exploited to improve the ATR performance.

#### 3.2.2. Binary Morphological Filtering of DSA Residue

**Figure 3:**
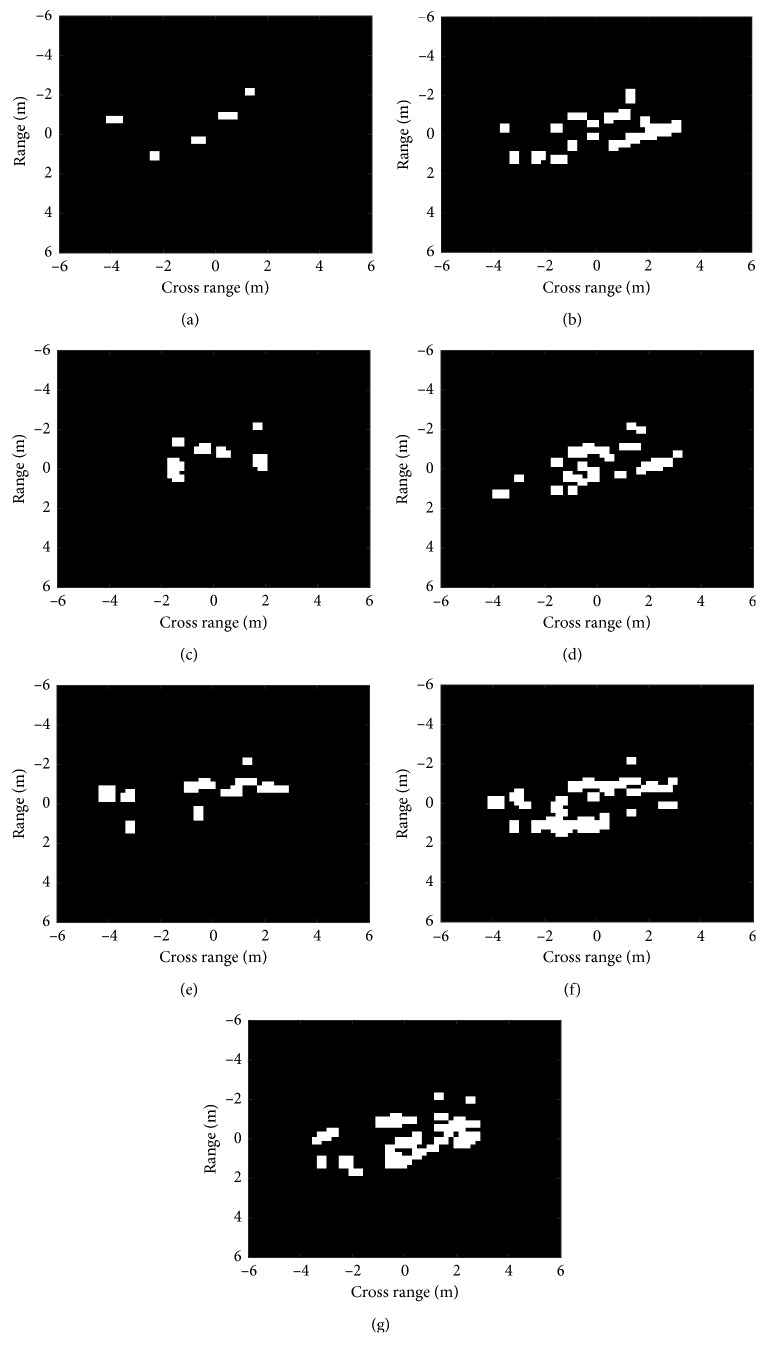
Filtered DSA residues between a BMP2 image and its corresponding templates from different classes: (a) BMP2 (Sn_9563); (b) BMP2 (Sn_9566); (c) BMP2 (Sn_c21); (d) BTR70; (e) T72 (Sn_132); (f) T72 (Sn_812); (g) T72 (Sn_s7).

The designed similarity measure in equation (4) mainly considers the area of the DSA residues but ignores the distributions of the residues. As shown in Figure 2, the residues within the same class are distributed in small patches. In contrast, the DSA residues between different classes gather together and are bulkily shaped. Therefore, the morphological opening operation is adopted to process the DSA residues to further enhance the differences in different classes. The opening operation using the structuring element of *S* is performed on the DSA residue *R* to eliminate the small patches as follows:

(5) $$\begin{array}{c} \text{RR}=R\;\circ \;S, \end{array}$$

where RR represents the filtered DSA residues after the opening operation. We set the structuring element as $S=\left[\begin{array}{cc} 1 & 1\\ 1 & 1 \end{array}\right]$ and then apply the opening operation to the DSA residues in Figure 2. The filtered DSA residues are shown in Figure 3. It is clear that the residues between same classes are significantly reduced, but the residues between different classes just shrink slightly. Compared with the results in Figure 2 intuitively, the opening operation makes it much easier to distinguish different targets.

The similarity measure in equation (4) is further imposed on the filtered residues. As showcased in Table 1, the similarities of the original DSA residues and filtered residues are calculated based on the results in Figures 2 and 3, respectively. The differences of the similarity sets before and after the opening operation are calculated to be 0.45 and 0.56, respectively. It indicates that the differences in the DSA residues are enhanced after the opening operation, which is beneficial for correctly classifying different classes of targets. Therefore, the similarities based on the filtered DSA residues can better distinguish different kinds of classes to improve the ATR performance.

### 3.3. Score-Level Fusion

The choice of the structuring element has important influences on the filtered results. With little prior information about the distribution of DSA residues, this study uses several different structuring elements during the opening operation to handle the possible deformations of DSAs. Afterwards, a score-level fusion is employed to combine the similarities from different structuring elements.

Assume there are *M* different structuring elements *S*_*i*_(*i*=1,2,…, *M*) and their corresponding similarity sets are *f*_*m*_(*m*=1,2,…, *M*). For each *f*_*m*_, *f*_*m*_(*i*)(*i*=1,2,…, *C*) represents the similarity between the test image and the *i*th template class. A linear score-level fusion is performed as follows:

(6) $$\begin{array}{c} \text{fs}\left(i\right)=\omega_{1}f_{1}\left(i\right)+\omega_{2}f_{2}\left(i\right)+\cdots +\omega_{M}f_{M}\left(i\right)\;\left(i=1,2,\ldots ,C\right), \end{array}$$

where *ω*_*m*_(*m*=1,2,…, *M*) denotes the weight corresponding to the *m*th structuring element and fs(*i*) is the fused similarity between the test sample and *i*th template class. When different weight vectors are used, different structuring elements are assigned with different importance. With little prior information, all the weights are set to be equal, i.e., 1/*M*, in this study. After the fused similarities are calculated, the target label of the test image is decided to be the class with the highest similarity.

**Figure 4:**
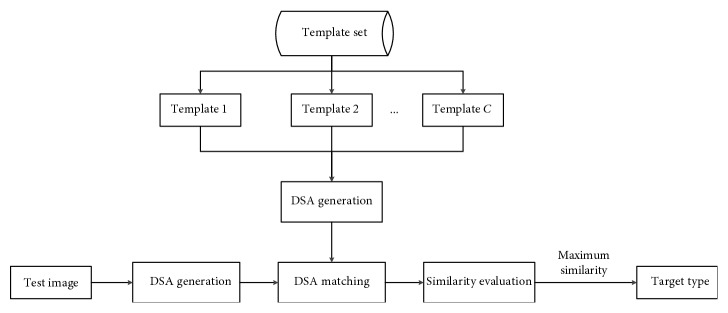
Procedure of the proposed target recognition method.

Figure 4 shows the general procedure of the proposed method. The azimuth of the test image is first estimated [28, 41] to obtain the corresponding template images from C target classes. Afterwards, the DSAs of the test image and the template images are generated at the scattering center number of 150. The DSAs are matched using the proposed method, and the score-level fusion is used to produce the final similarity. Finally, the target type of the test sample is assigned to the template class with the maximum similarity. The MSTAR images are well aligned, so the proposed method can be directly implemented on them. However, for images which are not aligned, some processing steps can be used to align them before the classification, such as target centralization [9].

## 4. Experiment

### 4.1. Data Preparation

**Figure 5:**
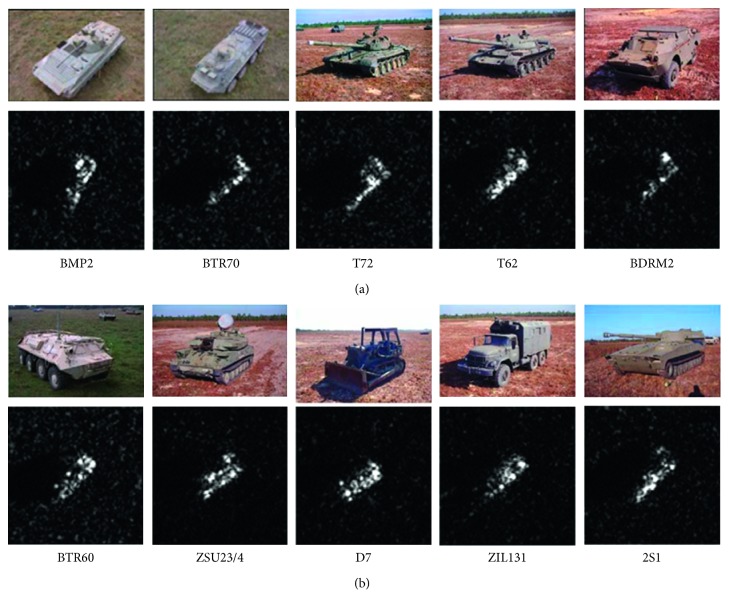
Types of military targets: (a) optical images versus (b) SAR images.

The MSTAR dataset is used to evaluate the performance of the proposed method, which includes SAR images of ten classes of ground targets: BMP2, BTR70, T72, T62, BDRM2, BTR60, ZSU23/4, D7, ZIL131, and 2S1. The optical images and exemplar SAR images of these targets are shown in Figure 5.  Table 2 showcases the detailed descriptions of the template and test sets. The template samples are collected at the 17° depression angle, whereas the test samples are at 15°. For quantitative evaluation, several state-of-the-art SAR ATR algorithms are used for comparison as described in Table 3. For simplicity, each of these methods is given an abbreviation according to the used feature or classifier. The Zernike and EFS methods both perform on the binary target regions, which first extract features from the binary target regions and employ SVM for classification afterwards. For SVM and SRC, they are adopted to classify the 80-dimension feature vectors from the original images extracted by PCA. CNN is directly trained by the intensity values of the original images. In the following sections, we first examine the proposed method under the standard operating condition (SOC) including the 3-class recognition problem and 10-class recognition problem. Afterwards, several typical EOCs are used to comprehensively evaluate the proposed method.

### 4.2. Experiments under SOC

#### 4.2.1. 3-Class Problem

A 3-class problem is first considered under SOC. The samples of BMP2, BTR70, and T72 at the 17° depression angle are used as the templates, and the samples at 15° are classified in Table 2. The structuring element is first set as $S=\left[\begin{array}{cc} 1 & 1\\ 1 & 1 \end{array}\right]$.  Table 4 presents the recognition results of the proposed method for 3-class recognition. Each of the configurations of three targets can be with a PCC (probability of correct classification) over 96%, and the average PCC is calculated to be 97.88%. The results indicate the high effectiveness of the proposed method in this condition.

The structuring element directly influences the filtered DSA residues. Therefore, the recognition performance is closely related to the choice of the structuring elements. As a further validation, we use several different structuring elements for filtering the DSA residues, which are listed as follows:

(7) $$\begin{array}{c} S_{1}=\left[\begin{array}{cc} 1 & 0\\ 0 & 1 \end{array}\right],\\ S_{2}=\left[\begin{array}{cc} 1 & 1\\ 1 & 1 \end{array}\right],\\ S_{3}=\left[\begin{array}{cc} 1 & 1\\ \text{0} & \text{0}\\ 1 & 1 \end{array}\right],\\ S_{4}=\left[\begin{array}{ccc} 1 & 0 & 1\\ 1 & 0 & 1 \end{array}\right],\\ S_{5}=\left[\begin{array}{ccc} 1 & 1 & 1\\ 1 & 1 & 1\\ 1 & 1 & 1 \end{array}\right]. \end{array}$$

The average PCCs at different structuring elements are showcased in Table 5. When the structuring element is null, i.e., no opening operation is performed, the PCC is the lowest. The PCC at *S*_5_ is lower than those at *S*_1_, *S*_2_, *S*_3_, and *S*_4_ mainly because *S*_5_ is a very large structuring element, so it removes too many pixels in the DSA residues from the incorrect classes. As a result, the differences in filtered DSA residues would not be notable enough for the high-performance target recognition.

To further enhance the effectiveness and robustness of the proposed method, the results of different structuring elements are fused by the score-level fusion. The fused results at different combinations of the structuring elements are compared in Table 6. The best performance of the score-level fusion is achieved at the combination of *S*_1_, *S*_2_, *S*_3_, *S*_4_ with the average PCC of 98.42%. Accordingly, this combination is used for target recognition in the following experiments.

The proposed method is compared with other methods in Table 7. The proposed method achieves the highest PCC. Compared with the Zernike and EFS methods, where the binary target regions are used as the baseline features, the proposed method outperforms them significantly. Moreover, the proposed method does not need further feature extraction from the binary target regions. CNN achieves an approaching PCC with the proposed method. The classification capability of CNN is closely related to the completeness and coverage of the training set. In this experimental setup, there are some configuration differences between the training and test samples of BMP2 and T72. As a result, the average PCC of CNN is slightly lower than the proposed method.

#### 4.2.2. 10-Class Problem

**Figure 6:**
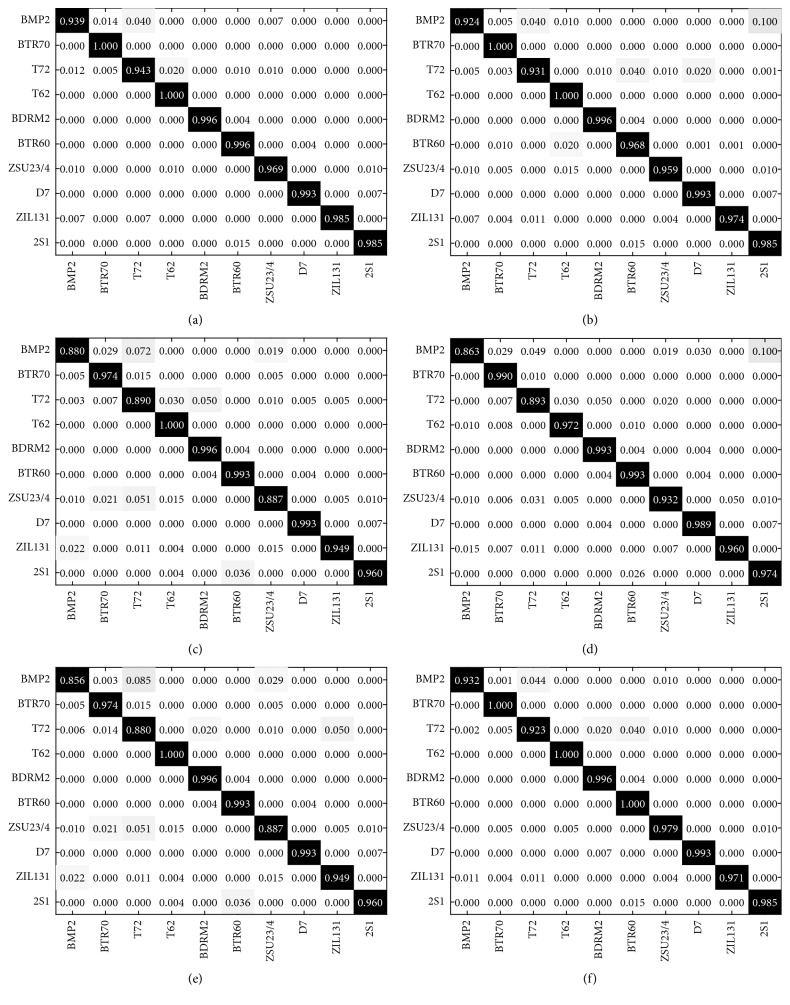
Confusion matrices of different methods under SOC: (a) proposed method; (b) Zernike; (c) EFS; (d) SVM; (e) SRC; (f) CNN.

A more challenging task, i.e., the 10-class recognition problem, is conducted in this part. All the 10-class samples listed in Table 2 are utilized in this experiment.  Table 8 showcases the detailed recognition results of the proposed method for the 10-class recognition. BMP2 and T72 suffer the lowest PCCs due to the configuration variances between their test and template sets.  Figure 6 shows the confusion matrices of the reference methods for detailed comparison. The average PCCs of different methods are compared in Table 9. All the methods experience some degradations in this case because the classification of 10 classes of targets is more difficult than the 3-class recognition problem. With the highest PCC of 97.24%, the proposed method still outperforms the others under 10-class recognition.

### 4.3. Experiments under EOC

The main obstacles to SAR ATR are the various EOCs caused by the variations of the target, SAR sensors, background environments, etc. Therefore, it is desired that the proposed method could achieve good robustness under different types of EOCs.

#### 4.3.1. Configuration Variance

For a certain target, it may have several configurations for different applications. In this case, it is desired that the ATR methods could keep robustness under configuration variance.  Table 10 lists the template and test sets in this experiment, in which the test configurations of BMP2 and T72 are different from those of their template samples.  Table 11 compares the average PCCs of different methods. With the highest PCC, the proposed method is validated to be the most robust to configuration variance. Under configuration variance, some local structures of the target are modified, whereas the whole target shape remains stable. As a result, the intensity distribution of the whole image may change greatly due to the local differences. The methods based on the image intensities including SVM, SRC, and CNN degrade significantly. In comparison, the methods using the binary target regions like Zernike and EFS achieve relatively better performance because the target shape remains stable. For the proposed method, the preliminary target shape is reflected by DSA. In addition, the small modifications in the local scattering centers can also be handled by the morphological opening operations with different structuring elements. Therefore, the proposed method can maintain its good performance under configuration variance.

#### 4.3.2. Large Depression Angle Variance

**Figure 7:**
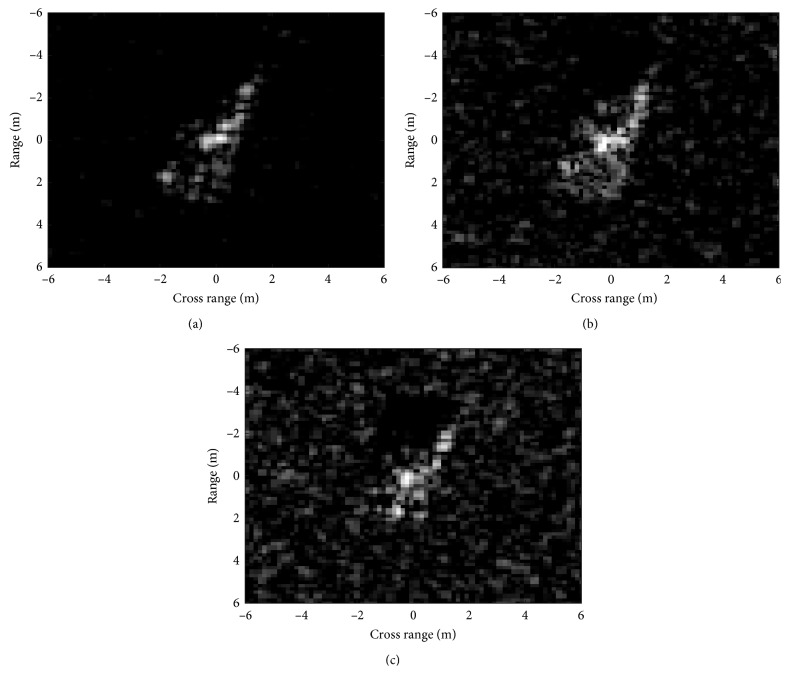
SAR images at depression angles of (a) 17°; (b) 30°; (c) 45°.

The SAR platforms may operate at different heights from the ground. As a result, the depression angles of the real-measured images may be quite different [42]. Actually, the template samples may be collected at only one or few depression angles. So, it is important that the proposed method could work robustly when the test and template images have large depression angle variance. Three targets are used in this experiment as showcased in Table 12, i.e., 2S1, BDRM2, and ZSU23/4. Their images at the 17° depression angle are used as the template set, whereas images at 30° and 45° are classified.  Figure 7 illustrates the differences in the SAR images at different depression angles with the images of 2S1 at 17°, 30°, and 45°, respectively. The figures show that both the target shape and scattering pattern vary when the depression angle changes. The recognition results of different methods are compared in Table 13. At the depression angle of 30°, the average PCCs of all the methods still remain at high levels. However, when the depression angle changes to 45°, the performance of different methods decreases sharply because the targets' appearances are notably different on comparing Figures 7(a) and 7(c). The proposed method can better cope with the nonlinear deformations of the DSA caused by large depression angle variance than the Zernike and EFS methods, which directly extract features from the binary target regions. For SVM, SRC, and CNN, their performance degrades most severely due to the drastic variation of the image intensity distribution. With the highest PCCs at both 30° and 45°, the proposed method is demonstrated to be the most robust to large depression angle variance.

#### 4.3.3. Noise Corruption

**Figure 8:**
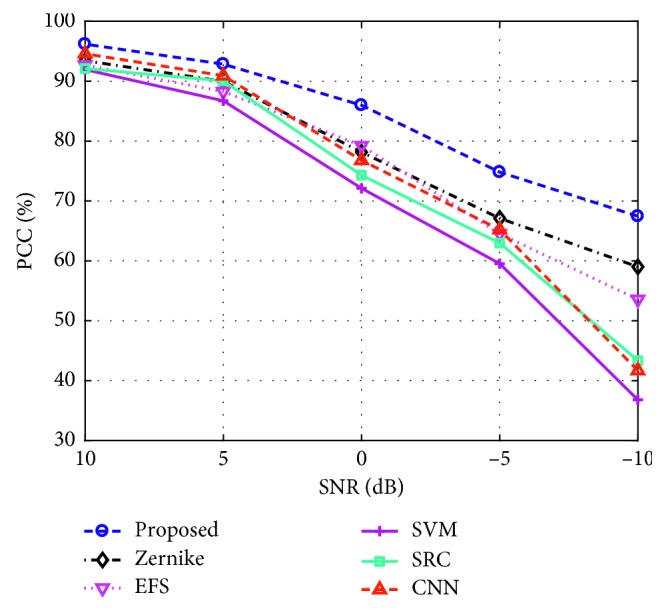
Performance of different methods under different levels of noise corruption.

The original SAR images in the MSTAR dataset are collected at high SNRs (signal-to-noise ratios), which is assumed to be an important reason for the excellent performance of different SAR ATR methods under SOC [43]. However, in practical applications, the real-measured SAR images may be contaminated by the noises from the background environment or radar system [43–45]. Then, it is probable that the test samples have much lower SNRs than the template ones, which are usually collected under some cooperative conditions. In this study, the noisy test samples are simulated by adding different levels of Gaussian noises to the original 10-class test samples in Table 2. In detail, the original SAR image is first transformed into the frequency domain using the inverse fast Fourier transform (IFFT). Afterwards, the additive Gaussian noises are added to the frequency spectrum according to the desired SNR. Finally, the noise-contaminated frequency data are transformed back into the image domain to obtain the noisy samples.  Figure 8 shows the performance of different methods at different noise levels. Clearly, the proposed method remains to be the most robust under noise corruption with the highest PCC at each SNR. Although the whole image intensity distribution is corrupted by noises, the dominant scattering centers with the higher intensities can still remain relatively stable. Therefore, the DSAs can still be constructed with good precision, so the proposed method can work robustly under noise corruption. Similarly, the binary target region is more robust than the whole image intensities or PCAs features. Therefore, the Zernike and EFS methods outperform the remaining ones.

#### 4.3.4. Partial Occlusion

**Figure 9:**
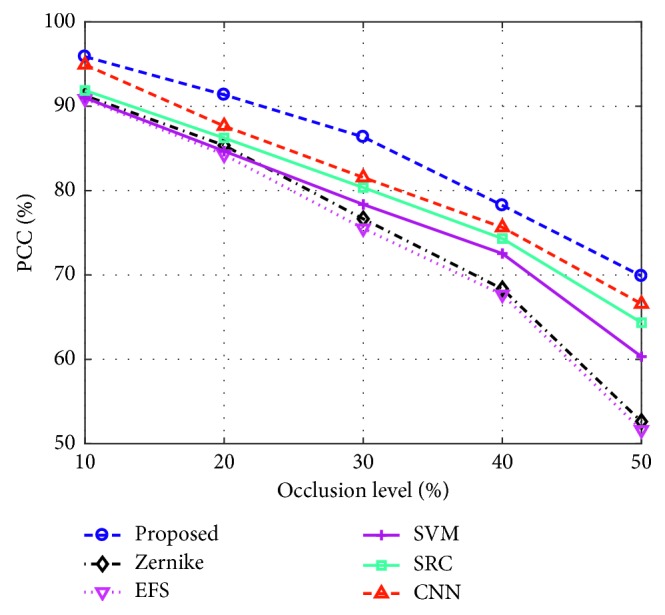
Performance of different methods under different levels of partial occlusion.

It is common that the ground targets are occluded by the natural or man-made obstacles, e.g., trees and buildings. As a result, some of the target characteristics cannot be collected by the sensors. For experimental evaluation, the partially occluded SAR images are first constructed according to the occlusion model in [46, 47]. In detail, a proportion of the original target regions is replaced by the background pixels from different directions. Afterwards, the occluded samples are classified by different methods.  Figure 9 plots the performance of all the methods at different occlusion levels. When the target is partially occluded, the extracted target shape probably deforms greatly. As a result, the methods solely depending on the binary target regions, i.e., Zernike and EFS, degrade significantly. For the proposed method, the highest PCC remains at each occlusion level because of the following reasons: on the one hand, the distribution of the dominant scattering centers is still discriminative although some scattering centers are occluded. On the other hand, the morphological opening operations using different structuring elements can effectively handle the possible deformations of DSA caused by partial occlusion.

## 5. Conclusion

An SAR ATR method is proposed via binary morphological filtering of the DSA residues. The DSA residues between the test image and its corresponding template images are processed by the binary morphological opening operation. Afterwards, a similarity measure is designed based on the filtered DSA residues for target recognition. To further improve the effectiveness and robustness of the proposed method, the morphological opening operations are conducted at different structuring elements, whose results are combined by a score-level fusion. Finally, the target label of the test sample is decided to be the class with the highest fused similarity. According to the experimental results on the MSTAR dataset, several conclusions can be drawn as follows: (1) under SOC, the proposed method achieves very high PCCs of 98.83% and 97.38% for 3-class and 10-class problems, respectively. Therefore, the proposed method can be used to distinguish many types of ground targets with good performance. (2) The proposed method remains the most robust under EOCs. (3) In comparison with several state-of-the-art SAR ATR methods, the proposed method outperforms them on both the effectiveness and robustness. So, it has much potential in the practical applications of SAR ATR.

Some future works are as follows: on the one hand, the numbers of the dominant scattering points should be decided adaptively for different types of targets such as ground targets, ships, and airplanes. On the other hand, more effective decision fusion strategies can be adopted or designed to combine the results from different structuring elements to further enhance the ATR performance.

## Figures and Tables

**Table 1:** Similarities based on the original DSA residues and filtered residues.

Target class	BMP2 Sn_9563	BMP2 Sn_9566	BMP2 Sn_c21	BTR70	T72 Sn_132	T72 Sn_812	T72 Sn_s7
Original residues	0.66	0.39	0.59	0.35	0.41	0.21	0.22
Filtered residues	0.91	0.60	0.83	0.57	0.64	0.35	0.40

**Table 2:** Details of template and test sets of the ten classes of targets.

Class	BMP2	BTR70	T72	T62	BDRM2	BTR60	ZSU23/4	D7	ZIL131	2S1
Template set (17°)	233 (Sn_9563)	233	232 (Sn_132)	299	298	256	299	299	299	299
Test set (15°)	195 (Sn_9563) 196 (Sn_9566) 196 (Sn_c21)	196	196 (Sn_132) 195 (Sn_812) 191 (Sn_s7)	273	274	195	274	274	274	274

**Table 3:** Methods for comparison.

Abbreviation	Classifier	Feature	Reference
Zernike	SVM	Zernike moments	[9]
EFS	SVM	Elliptical Fourier series coefficients	[12]
SVM	SVM	PCA features	[26]
SRC	SRC	PCA features	[30]
CNN	CNN	Intensity values	[33]

**Table 4:** Recognition results of the proposed method on 3-class data.

Test samples	Results			PCC (%)
	BMP2	BTR70	T72	
BMP2Sn_9563 (195)	193	1	2	98.97
BMP2Sn_9566 (196)	189	4	3	96.43
BMP2Sn_c21 (196)	190	2	4	96.94
BTR70 (196)	0	195	1	99.49
T72Sn_132 (196)	2	0	194	98.98
T72Sn_812 (195)	2	3	191	97.45
T72Sn_s7 (191)	2	3	186	97.38
Average PCC (%)	98.02			

**Table 5:** Average PCCs achieved by different structuring elements.

Structuring element	∅	*S*_1_	*S*_2_	*S*_3_	*S*_4_	*S*_5_
PCC (%)	95.34	97.88	98.02	97.87	97.64	97.02

**Table 6:** The performance of the fusion of different structuring elements.

Combination of structuring elements	Average PCC (%)
*S*_2_, *S*_3_	98.12
*S*_2_, *S*_3_, *S*_4_	98.34
*S*_1_, *S*_2_, *S*_3_, *S*_4_	98.83
*S*_1_, *S*_2_, *S*_3_, *S*_4_, *S*_5_	97.94

**Table 7:** Recognition performance for the 3-class problem.

Method	Proposed	Zernike	EFS	SVM	SRC	CNN
PCC (%)	98.83	95.48	96.46	96.87	95.66	98.36

**Table 8:** Confusion matrix of the proposed method under SOC.

Class	BMP2	BTR70	T72	T62	BDRM2	BTR60	ZSU23/4	D7	ZIL131	2S1	PCC (%)
BMP2	**563**	8	15	0	0	4	5	0	3	0	95.91
BTR70	0	**196**	0	0	0	0	0	0	0	0	100
T72	8	4	**549**	0	6	0	4	4	1	5	94.49
T62	0	0	0	**273**	0	0	0	0	0	0	100
BDRM2	0	0	0	1	**271**	0	1	0	1	0	98.91
BTR60	0	1	0	1	0	**190**	0	3	0	0	97.44
ZSU23/4	0	1	0	0	1	0	**269**	0	0	3	98.18
D7	0	0	0	0	0	0	0	**274**	0	0	100
ZIL131	3	1	1	0	0	0	1	0	**268**	0	97.81
2S1	0	0	0	1	0	1	0	0	0	**272**	99.27
Average											97.38

**Table 9:** Average PCCs of different methods for the 10-class problem.

Method	Proposed	Zernike	EFS	SVM	SRC	CNN
PCC (%)	97.38	94.41	93.97	94.22	93.66	97.24

**Table 10:** Template and test datasets with configuration variance.

	BMP2	T72	BTR60	T62
Template set (17°)	233 (Sn_9563)	232 (Sn_132)	256	299
Test set (15°)	196 (Sn_9566) 196 (Sn_c21)	195 (Sn_812) 191 (Sn_s7)	195	273

**Table 11:** Recognition performance on configuration variance.

Method	Proposed	Zernike	EFS	SVM	SRC	CNN
PCC (%)	95.25	93.24	92.92	89.72	90.14	92.66

**Table 12:** Template and test sets with large depression angle variance.

	Depression	2S1	BDRM2	ZSU23/4
Template set	17°	299	298	299
Test set	30°	288	287	288
	45°	303	303	303

**Table 13:** PCCs of different methods under different depression angles (%).

	Proposed	Zernike	EFS	SVM	SRC	CNN
30°	96.24	94.17	93.15	92.57	91.92	94.88
45°	73.54	63.23	61.16	56.96	60.27	63.14

## Data Availability

The MSTAR dataset used to support the findings of this study is available online at http://www.sdms.afrl.af.mil/datasets/mstar/.
